# Risk stratification model for foreseeing overall survival in Chinese patients with initially metastatic small-cell lung cancer

**DOI:** 10.1097/MD.0000000000040145

**Published:** 2024-10-18

**Authors:** Rong Fu, Chuanqing Jing, Wei Zhang

**Affiliations:** a The First Clinical Medical College of Shandong University of Traditional Chinese Medicine, Jinan, China; b Affiliated Hospital of Shandong University of Traditional Chinese Medicine, Jinan, China.

**Keywords:** Chinese patients, nomogram, overall survival, risk stratification, small-cell lung cancer

## Abstract

The study was outlined to develop and approve a nomogram and chance stratification demonstrate for foreseeing overall survival of Chinese patients with at first metastatic small-cell lung cancer (SCLC). We collected information from the Surveillance, Epidemiology, and End Results (SEER) database approximately Chinese SCLC patients with at first distant metastases between 2010 and 2015. Patients with incomplete data about the follow-up time or clinicopathological information were excluded. The included patients were randomized into the training and validation set. Univariate and multivariate Cox proportional hazard regression models were performed. By integrating the significant variables screened, a prescient nomogram and risk stratification model were developed. In addition, we collected 198 small-cell lung cancer patients with metastasis at diagnosis from the case database of the Affiliated Hospital of Shandong University of Traditional Chinese Medicine as an external validation cohort. In all, 421 patients were screened from the SEER database. Multivariate examination showed that age (*P* = .049), sex (*P* = .001), grade (*P* = .008), chemotherapy (*P* = .001), liver metastasis (*P* = .001), and pleural invasion (*P* = .012) were independent prognostic factors. The C-indicator of the nomogram to anticipate overall survival was higher than that of the eighth edition of the American Joint Committee on Cancer Tumor Node Metastasis classification system (0.75 vs 0.543; *P* < .001). A risk stratification model was encouraged to be created to precisely classify patients into 2 prognostic bunches. The survival rates anticipated by the nomogram appeared to have critical qualifications from the Kaplan–Meier curves in the entire SEER cohort. Calibration curves and survival predictions also showed strong accuracy and consistency in the external validation cohort. The nomogram provided a clear prognostic superiority over the traditional Tumor Node Metastasis system. It could help clinicians make individual risk predictions for initially metastatic Chinese SCLC cancer patients and give necessary treatment recommendations.

## 1. Introduction

Lung cancer is the driving cause of cancer death rate. Approximately 14% of all lung cancer cases in the United States (more than 30,000 patients) have small-cell lung cancer (SCLC).^[1]^ Chemotherapy is still the first-line treatment for SCLC, but most patients still relapse within a year of treatment due to drug resistance.^[2,3]^ With minimal breakthroughs that occurred in the past decade, the therapeutic success remains unsatisfactory. In spite of a tall beginning reaction to treatment, most patients pass on from repetitive malady, and the middle survival after conclusion is evaluated to be 8 to 20 months. Moreover, although immunotherapy offers options for the treatment of extensive-stage SCLC, there are fewer drugs available and the benefits are more limited. Radiotherapy in extensive stages is an important therapeutic strategy to delay progression. In addition, there are not many targeted treatment options due to the lack of driver targets. Surgery is not indicated for metastatic SCLC.^[4]^

The eighth edition of the American Joint Committee on Cancer Tumor Node Metastasis (AJCC-TNM) staging is the most commonly used tool for assessing the survival prognosis of patients with SCLC, but the main limitations are related to the neglect of other factors (e.g., demographic characteristics and tissue characteristics) and the poor predictive performance of individual prognostic risk.^[5]^ In recent years, nomograms have gained increasing attention as strong prognostic statistical models with intuitive graphs to quantify risks by incorporating factors important for oncologic prognosis.^[6–8]^ They are useful and convenient tools to estimate the risk and prognosis in cancer patients. For lung cancer patients, a number of prognostic nomograms have been developed and validated based on traditional clinicopathological features. Furthermore, the majority of these models were developed for Caucasian patients rather than for Asian patients. At present, there are few nomograms for Chinese patients with metastatic SCLC.

Hence, in this study, we combined the Surveillance, Epidemiology, and End Results (SEER) data with our population, incorporating many factors such as demographic information characteristics, tumor histological characteristics, treatment information, and variables in TNM staging, to establish and approve a nomogram for the prognosis of Chinese SCLC patients.

## 2. Material and methods

### 2.1. Study design and patients selection

**Figure 1.**
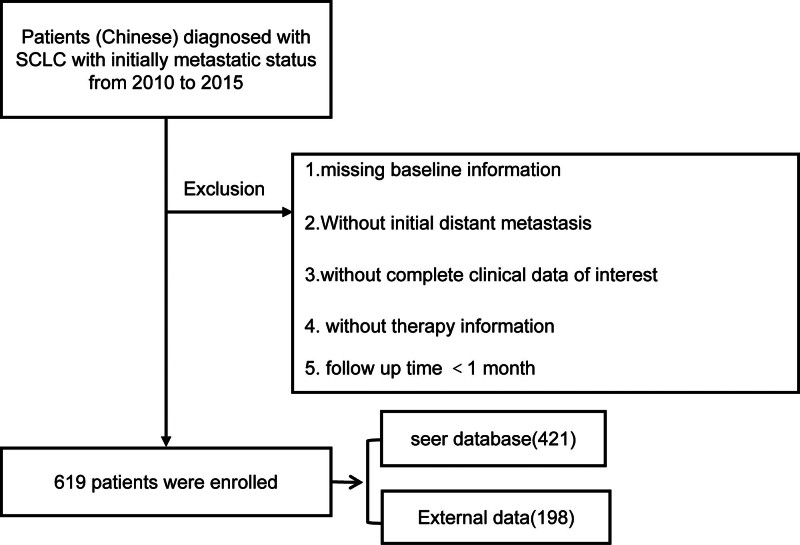
Flowchart of the sample selection. SCLC = small-cell lung cancer.

By utilizing SEER*stat adaptation 8.3.5, we selected 2209 patients with metastatic SCLC in the period between 2010 and 2015. The inclusion criteria were as follows: patients with histologically confirmed SCLC; year of diagnosis ranging from 2010 to 2015; age ≥ 18 years at the time of diagnosis; primary tumor in the lung; and first distant metastasis and no surgery. Exclusion criteria were as follows: patients with incomplete baseline information. For example, lack of data on tumor stage, histological grading, tumor size, tumor site, age, time of diagnosis, and marital status; patients with incomplete or no record of treatment information during follow-up; distant metastasis was not the first occurrence; and follow-up time < 1 month. Eventually, we collected the age at diagnosis, gender, marital status, tumor size, histological grading, tumor location, TNM stage (including T stage and N stage), distant metastasis (i.e., M1 stage), radiotherapy, and follow-up time. Among them, according to the guidelines for the diagnosis and treatment of SCLC, we classified the tumor diameter into 2 statistical subgroups, <1 and ≥1, due to the faster growth and invasion of SCLC tumors, and the survival rate of T1a stage was significantly higher than that after T1a stage.^[9]^ For World Health Organization histologic grading, both its and TNM staging were highly correlated with patient survival. Age at diagnosis, gender, marital status, and tumor location responded to the disease base of the patient, while radiotherapy status and follow-up time reflected the treatment information and survival time of the patient. Distant metastasis was selected as a variable with reference to the metastatic site of positive M1 stage in TNM staging. The selection flowchart is shown in Figure 1. Finally, 1788 patients were excluded, and 421 eligible patients were enrolled into the study and randomly partitioned in the training cohort (70%, n = 297) and internal validation cohort (30%, n = 124).^[10]^ For this type of retrospective study, there is no need for obtaining formal consent from the patients. SCLC patients with metastases at their first visit from 2010 to 2015 were collected in the medical record system of the Affiliated Hospital of Shandong University of Traditional Chinese Medicine and evaluated according to the same criteria, resulting in data from 198 cases.

### 2.2. Statistical methods

A single-variable comparison of survival information was performed utilizing the Kaplan–Meier strategy and Cox univariate examination. According to the comes about of the single-variable examination (*P* < .1) and combined with clinically critical variables, a multivariate examination utilizing the Cox hazard relapse show with in reverse disposal was performed. After the multivariate examination, factors with *P* < .05 were chosen for creating the nomograms. Sensitivity analyses were also performed with continuous multivariate Cox regression analyses in externally validated data collected at our institution.

We utilized 1-, 2-, and 3-year overall survival (OS) for the examination within the nomogram. In addition, we calculated the C-index using bootstrapping (1000 resamplings) and plotted calibration plots to assess the accuracy of the nomograms. Moreover, a chance stratification demonstration was created on the premise of each patient’s add-up score within the nomogram to partition all cases into 2 prognostic bunches concurring to its middle esteem.

All of the investigations were performed utilizing R (http://www.r-project.org) and Engage (R) (www.empowerstats.com, XY Arrangements, Inc., Boston). Statistical significance was confirmed if *P* was lower than .05 in a 2-tailed test.

## 3. Results

### 3.1. Characteristics of patients

A total of 421 patients were recognized from the SEER database in agreement with the screening inquiries (Fig. 1). The middle follow-up time for the whole cohort was 7 months.

The 1-, 2-, and 3-year survival rates were 0.37, 0.16, and 0.07, respectively. We randomly assigned 70% (n = 297) of the entire cohort to the training cohort and 30% (n = 124) to the internal approval cohort. Demographic and clinicopathological characteristics are concentrated in Table 1. Basic information about the external data we collected is also shown in Table 1.

**Table 1:** Patients demographic and clinicopathological characteristics.

Factors	SEER cohort (n = 421)		Training cohort (n = 297)		Validation cohort (n = 124)		External cohort (n = 198)	
	N	%	N	%	N	%	N	%
Age at diagnosis, yr								
Median	72		72		72		71	
Range	30–95		30–93		30–95		30–95	
Sex								
Male	227	53.9	151	50.8	76	61.3	109	55.1
Female	194	46.1	146	49.2	48	38.7	89	44.9
YOD								
2010–2012	174	41.3	118	39.7	56	45.2	83	41.9
2013–2015	247	58.7	179	60.3	88	54.8	115	58.1
Marital status								
Married	307	72.9	210	70.7	97	78.2	169	85.4
Not married	114	27.1	87	29.3	27	21.8	29	14.6
Tumor size, diameter, cm								
<1	191	45.4	139	46.8	52	41.9	85	42.9
≥1	230	54.6	158	53.2	72	58.1	113	57.1
Histological grade								
Well differentiated	29	6.9	23	7.7	6	4.8	13	6.7
Moderately differentiated	141	33.5	95	32.0	46	37.1	72	36.4
Poorly differentiated	237	56.3	170	57.2	67	54.0	109	55.1
Undifferentiated	14	3.3	9	3.0	5	4.0	4	1.8
Laterality								
Left	174	41.3	125	42.1	49	39.5	86	43.4
Right	247	58.7	172	57.9	75	60.5	112	56.6
T								
T1	27	6.4	18	6.1	9	7.3	26	12.6
T2	121	28.7	81	27.3	40	32.3	64	32.3
T3	122	29.0	88	29.6	34	27.4	51	25.8
T4	151	35.9	110	37.0	41	33.1	57	29.3
N								
N0	111	26.4	80	26.9	31	25	49	24.7
N1	28	6.7	16	5.4	12	9.7	10	5.1
N2	187	44.4	133	44.8	54	43.6	96	48.5
N3	95	22.6	68	22.9	27	21.8	43	21.7
Radiotherapy								
No	242	57.5	179	60.3	63	50.8	115	58.1
Yes	179	42.5	118	39.7	61	49.2	83	41.9
Chemotherapy								
No	153	36.3	108	36.4	45	36.3	77	38.9
Yes	268	63.7	189	63.6	79	63.7	121	61.1
Pleural invasion								
No	380	90.3	271	91.3	109	87.9	182	91.9
Yes	41	9.7	26	8.7	15	12.1	16	8.1
Bone metastasis								
No	248	58.9	177	59.6	71	57.3	125	63.1
Yes	173	41.1	120	40.4	53	42.7	73	36.9
Brain metastasis								
No	312	74.1	230	77.4	82	66.1	157	79.2
Yes	109	25.9	67	22.6	42	33.9	41	20.8
Liver metastasis								
No	354	84.1	247	83.2	107	86.3	163	82.3
Yes	67	15.9	50	16.8	17	13.7	35	17.7
Lung metastasis								
No	278	66.0	192	64.7	86	69.3	134	67.7
Yes	143	34.0	105	35.3	38	30.7	64	32.3
Follow-up, mo								
Median	7		7		8		7	

YOD = year of diagnosis.

The median age at diagnosis for the entire SEER database population cohort was 72 years (interquartile range [IQR]: 30–95), with the training cohort being 72 (IQR: 30–93) and the validation cohort being 72 (IQR: 30–95). For follow-up time, it was 7 months for both the SEER cohort and the training cohort, and it was 8 months for the validation cohort. Regarding the basic characteristics of the affected population, male, married patients were in the majority throughout the SEER cohort, the training cohort, and the validation cohort. In terms of tumor characteristics, organization, and staging features, the majority of patients had tumors larger than 1 cm in size and were mostly distributed on the right side. The low-differentiation status population was significantly higher in the SEER cohort (56.3%), the training cohort (57.2%), and the validation cohort (54.0%) than in the other differentiation statuses. In the SEER cohort, it was higher in T4 (35.9%) and N2 (44.4%) stage patients. Again, this was proportionally similar in the training cohort (T4: 37.0%; N2: 44.8%) as well as the validation cohort (T4: 33.1%; N2: 43.6). In addition, in terms of distant metastasis, the proportion of pleural invasion was low in the entire population (SEER cohort: 9.7%; training cohort: 8.7%; validation cohort: 12.1%; and external cohort: 8.1%). Regarding treatment, chemotherapy was chosen by relatively more people in all 4 cohorts (SEER cohort: 63.7%; training cohort: 63.6%; validation cohort: 63.7%; and external cohort: 61.1%). The population characteristics of the external cohort were similar to those of the 3 cohorts in the SEER database.

### 3.2. Independent prognostic variables within the training and external cohort

Univariate Cox regression analysis illustrated that age at diagnosis, sex, conjugal status, histological review, chemotherapy, liver metastasis, and pleural invasion were related to by and large survival. All of these factors were, thus, entered into the multivariate Cox regression analysis. Age at diagnosis, sex, histological grade, chemotherapy, liver metastasis, and pleural invasion were found to be independent prognostic variables after the multivariate investigation (*P* < .05; Table 2). Sensitivity analyses showed no significant differences in independent risk factors between the external cohort and the training cohort (Table 3).

**Table 2:** Univariate and multivariate analysis in the training cohort.

Factors	Univariate analysis			Multivariate analysis			Score
	HR	95% CI	P*	HR	95% CI	P**	
Age at diagnosis, yr							
<72	1						0
≥72	1.8	1.4, 2.3	**<.001**	1.3	1.0, 1.6	**.049**	26
Sex							
Male	1						58
Female	0.5	0.4, 0.7	**<.001**	0.6	0.5, 0.8	**<.001**	0
YOD							
2010–2012	1						
2013–2015	1.0	0.8, 1.2	.724				
Marital status							
Not married	1						
Married	0.8	0.6, 1.0	**.098**	0.9	0.7, 1.2	.573	
Tumor size, diameter, cm							
<1	1						
≥1	1.0	0.8, 1.3	.779				
Histological grade							
Moderately differentiated	1						0
Well differentiated	1.1	0.7, 1.8	.752	1.1	0.7, 1.7	.794	33
Poorly differentiated	1.8	1.1, 2.8	**.017**	1.4	0.9, 2.2	.167	67
Undifferentiated	3.6	1.8, 7.5	**<.001**	2.7	1.3, 5.7	**.008**	100
Laterality							
Left	1						
Right	1.0	0.8, 1.3	.997				
T							
T1	1						
T2	1.1	0.7, 1.9	.611				
T3	1.5	0.9, 2.4	.141				
T4	1.3	0.8, 2.2	.258				
N							
N0	1						
N1	0.8	0.5, 1.4	.487				
N2	1.1	0.9, 1.5	.358				
N3	1.2	0.8, 1.6	.336				
Radiotherapy							
No	1						
Yes	1.0	0.8, 1.3	.997				
Chemotherapy							
No	1						97
Yes	0.3	0.3, 0.4	**<.001**	0.4	0.3, 0.5	**<.001**	0
Pleural invasion							
No	1						0
Yes	1.3	1.1, 1.8	**.019**	1.6	1.1, 2.3	**.012**	64
Bone metastasis							
No	1						
Yes	0.9	0.7, 1.2	.543				
Brain metastasis							
No	1						
Yes	1.1	0.9, 1.4	.450				
Liver metastasis							
No	1						0
Yes	1.8	1.3, 2.4	**<.001**	1.6	1.2, 2.2	**.001**	48
Lung metastasis							
No	1						
Yes	1.0	0.8, 1.3	.964				

95% CI = 95% confidence interval, HR = hazard ratio, YOD = year of diagnosis.

* *P* < .1 was considered significant in univariate Cox regression analysis.

** *P* < .05 was considered significant in multivariate Cox regression analysis.

**Table 3:** Univariate and multivariate analysis in external validation cohort.

	Univariate analysis			Multivariate analysis		
	HR	95% CI	P*	HR	95% CI	P**
Age at diagnosis, yr						
<72	1					
≥72	1.5	1.3, 1.9	**<.001**	1.3	1.1, 1.5	**.03**
Sex						
Male	1					
Female	0.5	0.4, 0.7	**<.001**	0.5	0.3, 0.7	**.05**
YOD						
2010–2012	1					
2013–2015	1.0	0.7, 1.4	.529			
Marital status						
Not married	1					
Married	0.7	0.6, 0.9	**.074**	0.8	0.6, 1.0	.698
Tumor size, diameter, cm						
<1	1					
≥1	1.0	0.8, 1.3	.821			
Histological grade						
Moderately differentiated	1					
Well differentiated	1.2	0.6, 1.7	.708	1.2	0.8, 1.8	.871
Poorly differentiated	1.7	1.1, 2.6	**.023**	1.5	1.0, 2.2	.176
Undifferentiated	3.4	1.5, 6.8	**<.001**	2.3	1.3, 4.9	**.006**
Laterality						
Left	1					
Right	1.1	0.9, 1.3	.903			
T						
T1	1					
T2	1.0	0.7, 1.9	.638			
T3	1.4	0.8, 2.3	.159			
T4	1.4	0.7, 2.2	.279			
N						
N0	1					
N1	0.8	0.4, 1.2	.501			
N2	1.1	0.8, 1.4	.379			
N3	1.2	0.9, 1.5	.328			
Radiotherapy						
No	1					
Yes	1.0	0.7, 1.3	.927			
Chemotherapy						
No	1					
Yes	0.4	0.4, 0.5	**<.001**	0.4	0.2, 0.6	**<.001**
Pleural invasion						
No	1					
Yes	1.3	1.1, 1.5	**.023**	1.6	1.1, 2.1	**.019**
Bone metastasis						
No	1					
Yes	0.8	0.5, 1.2	.535			
Brain metastasis						
No	1					
Yes	1.1	0.9, 1.4	.457			
Liver metastasis						
No	1					
Yes	1.8	1.3, 2.5	**<.001**	1.6	1.2, 2.1	**.002**
Lung metastasis						
No	1					
Yes	1.0	0.8, 1.2	.895			

95% CI = 95% confidence interval, HR = hazard ratio, YOD = year of diagnosis.

* *P* < .1 was considered significant in univariate Cox regression analysis.

** *P* < .05 was considered significant in multivariate Cox regression analysis.

### 3.3. Structure and proof of the nomogram

**Figure 2.**
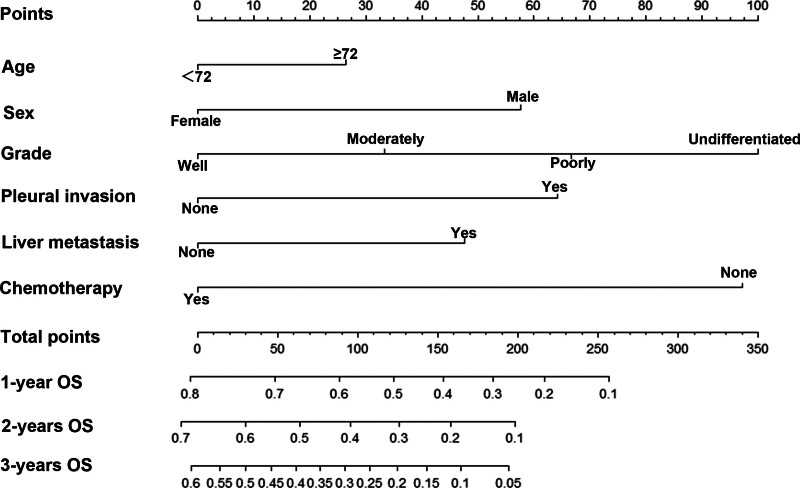
Nomogram anticipating 1, 2, and 3 years in general survival in Chinese patients with small-cell lung cancer. OS = overall survival.

For foreseeing the general survival of patients, these 6 significant separate variables were incorporated to construct a nomogram (Fig. 2). The score of each category was given on the point scale pivot (Table 2). The whole grade was effortlessly calculated by including each single grade and by anticipating the entire grade to the foot scale, where we can peruse the assessed probabilities of 1-, 2-, and 3-year OS for each patient.

**Figure 3.**
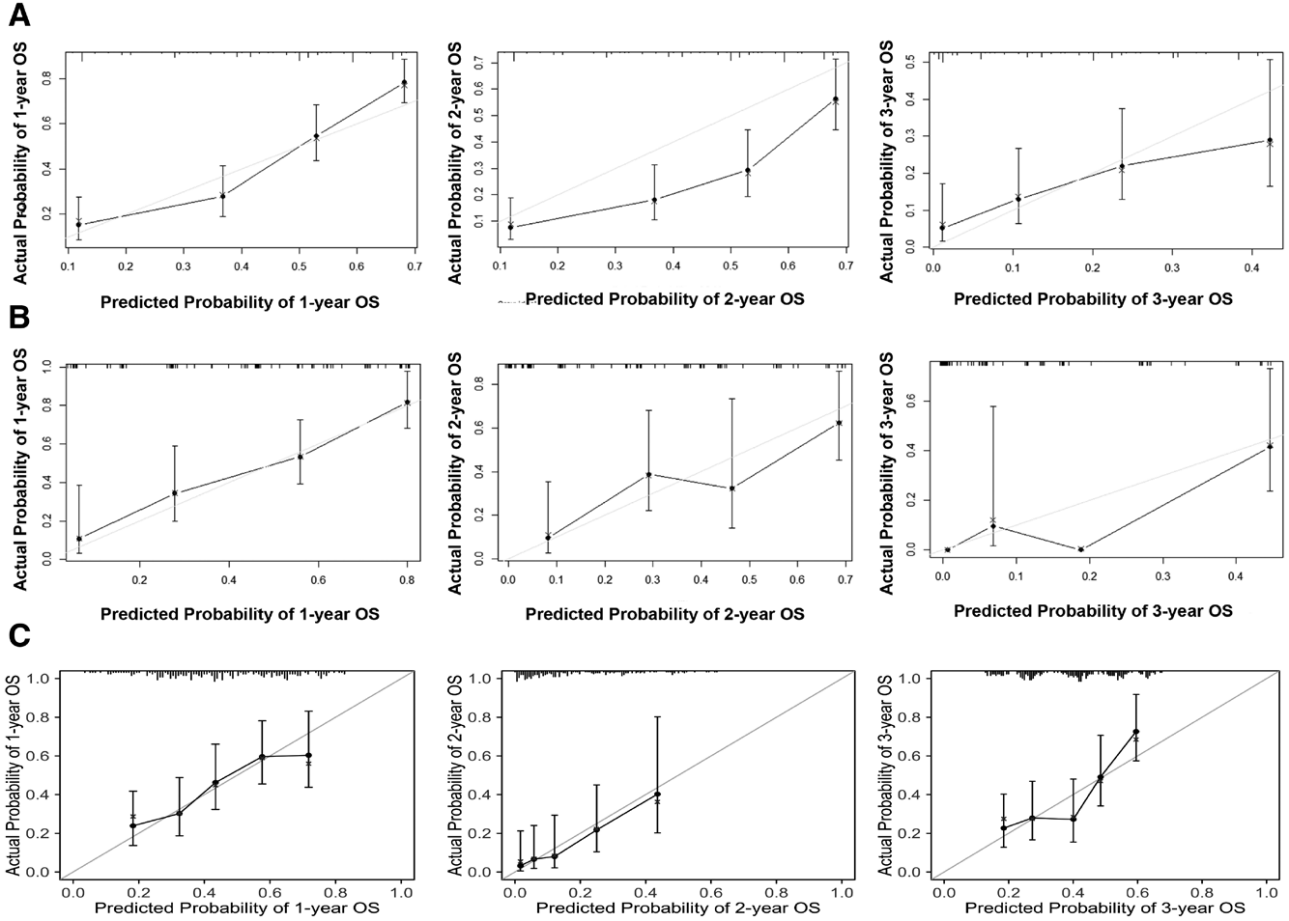
The calibration curves predicting 1-, 2-, and 3-year overall survival (OS) in the training cohort (A), validation cohort (B), and external cohort (C).

The C-index was bigger for the here-constructed nomogram than for the eighth form of the AJCC-TNM classification system (0.75 vs 0.543; *P* < .001), suggesting that this model had an acceptable predictive accuracy. In addition, we developed calibration plots of the column-line plots in the training cohort, validation cohort, and external cohort, respectively (Fig. 3), showing that the predicted OS rates were in good agreement with the actual observations.

**Figure 4.**
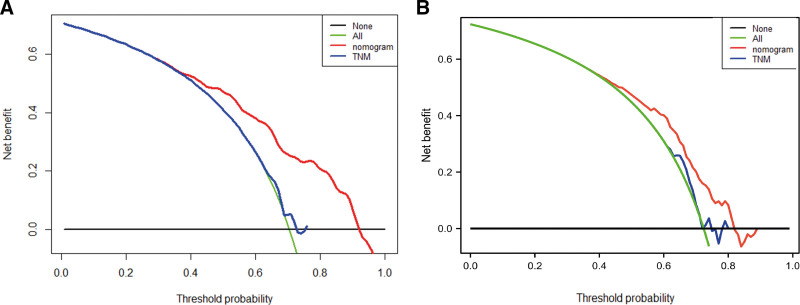
Decision curve analysis (DCA) comparing the performance of the nomogram and the Tumor Node Metastasis (TNM) staging model. (A) DCA of the training cohort. (B) DCA of the external cohort. The even dark line speaks to the presumption that no one ought to take the vital measures, whereas the green line speaks to the suspicion that all patients ought to take the essential measures. The y-axis speaks to the net advantage, which was calculated by including focuses related to benefits and subtracting those related to hurts. Based on the edge probabilities gotten, our discoveries show that the nomogram (ruddy line) has a more noteworthy net advantage than the TNM staging model (blue line).

Decision curve analysis could be a net advantage investigation that compares the true-positive to the weighted false-positive rates over distinctive hazard edges that a clinician/patient might need to acknowledge. This analysis was performed evaluating the 2-year OS of SCLC patients. As shown in Figure 4, all the models had a stronger net advantage compared to the “treat all” methodology. Moreover, the net benefit of the nomogram was higher than that of the TNM model in both the internal and external validation cohorts at a threshold range of 0.4 to 0.8, suggesting that the use of the nomogram to predict patient survival is more reliable than the TNM model for metastatic SCLC patients in China in most cases.

### 3.4. Risk stratification system

These results proved the nomograms’ efficacy for predicting survival in SCLC patients. Thus, we calculated total points in line accordance with the nomogram predicted score. The patients were partitioned into the low-risk group (total score, < 130.78) and high-risk group (total score, ≥ 130.78) according to the median points. Within the SEER cohort, 2-year OS rates in patients with low hazards and high hazards were 0.24 and 0.08, respectively. Consistently, in the external cohort, the 2-year OS rates were 0.31 and 0.12 for low-risk and high-risk patients.

**Figure 5.**
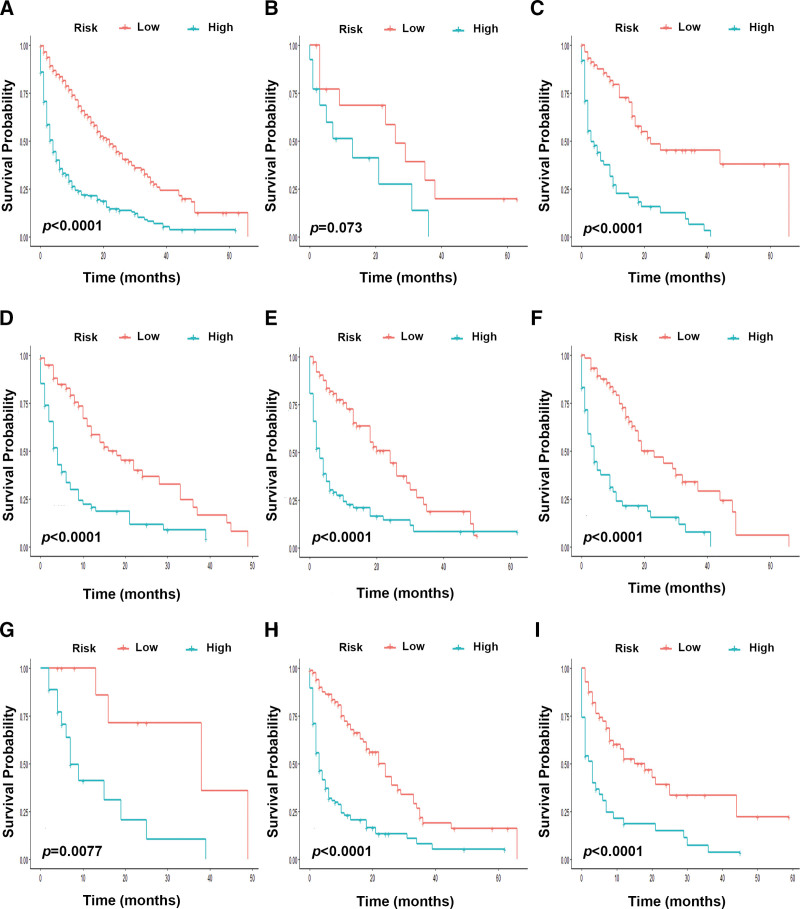
Risk stratification based on the nomogram. Kaplan–Meier curves for overall survival in the entire Surveillance, Epidemiology, and End Results cohort (A), T1 (B), T2 (C), T3 (D), T4 (E), N0 (F), N1 (G), N2 (H), and N3 (I) in small-cell lung cancer patients.

**Figure 6.**
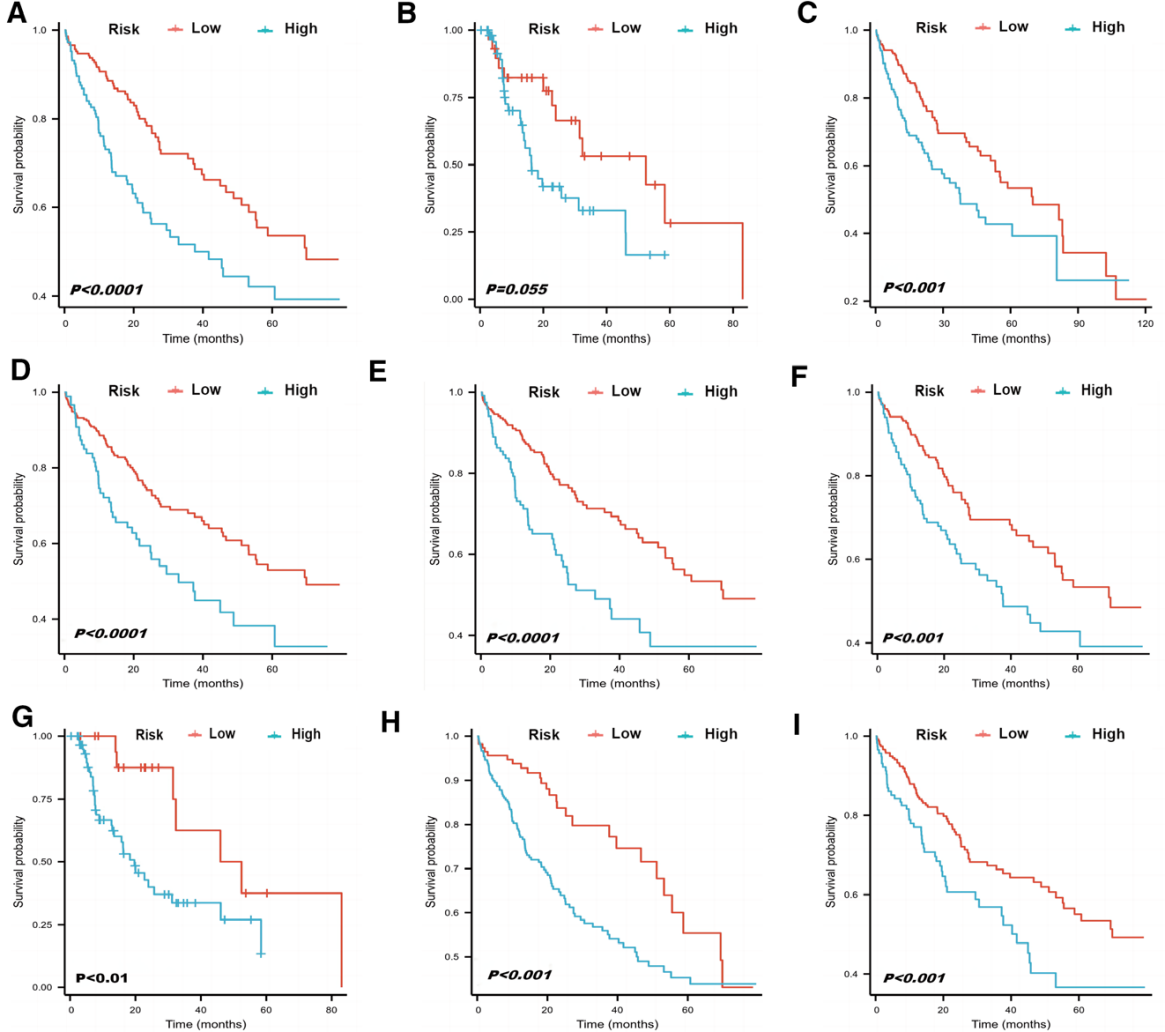
Risk stratification based on the nomogram. Kaplan–Meier curves for overall survival in the external cohort (A), T1 (B), T2 (C), T3 (D), T4 (E), N0 (F), N1 (G), N2 (H), and N3 (I) in small-cell lung cancer patients.

To approve the supplementary part of the risk stratification framework to the eighth version of the AJCC-TNM arrange framework, we stratified the training and external cohort based on the T and N classifications. Identically, there was no significant difference in T1 period (training cohort *P* = .073 vs external cohort *P* = .055). In addition, inside most of the T or N phases, the survival rates anticipated by the nomogram appeared noteworthy qualifications between Kaplan–Meier curves (Figs. 5A–I and 6A–I).

## 4. Discussion

The AJCC-TNM arranging classification is the foremost broadly utilized framework for foreseeing survival and choice of clinical procedures for patients with cancers.^[11,12]^ However, this classical framework does not always accurately identify the differences in survival between different stages.^[13]^ Furthermore, even patients with the same stage may show substantial variations in survival. Indeed, many factors confirmed to be highly associated with survival were often ignored in predicting survival. To solve this problem, we developed a nomogram as a more comprehensive, accurate, and useful prognostic model.^[14]^

Previous studies that established nomograms for foreseeing the survival of Chinese patients with initially metastatic SCLC had a small sample size and limited prognostic factors. In this way, we created a clinical nomogram to anticipate the survival based on the SEER database. At the same time, we included data from our hospital for external validation to evaluate the accuracy of the model. The SEER registry is the biggest population-based database of cancer patients in America, covering approximately 26% of patients diagnosed with cancer.^[15–17]^ We checked on patients’ information from the most recent form of the SEER that was discharged in 2015 (covering 18 registries, 1973–2015) by utilizing SEER*Stat form 8.3.5, and we also set strict inclusion and exclusion criteria.

Through univariate and multivariate Cox relapse analysis, we inevitably distinguished 6 free prognostic variables. They included age at diagnosis, sex, histological grade, chemotherapy, liver metastasis, and pleural invasion, all of which were finally incorporated in the clinical nomogram. These discoveries were reliable with past considers on indicators of survival in SCLC patients, but not identical. Clearly, our nomogram showed that the tumor’s histological grade category had the largest effect on the prognosis. Specifically, poorly differentiated tumors were highly associated with poor prognosis in Chinese SCLC patients. This is especially important for further treatment after obtaining puncture pathology results or after obtaining tissue in the surgical setting.

SCLC may be an exceptionally forceful danger characterized by tall cellular expansion and early metastatic spread. SCLC is also a chemosensitive and radiosensitive illness, in spite of the fact that the initial reaction to chemotherapy is ordinarily followed by the development of tolerance and a poor prognosis with a meddle survival time of <12 months.^[18–20]^ Furthermore, no significant progress in treatment of SCLC has been made over the recent years, and there have been no recently endorsed drugs. For all these reasons, SCLC speaks to a major challenge for oncologists and an energizing field for clinical investigation. In this study, we found that chemotherapy was an effective measure to improve the outcomes in Chinese SCLC patients; however, radiotherapy was not a free prognostic figure in this set of patients.

Our study also showed a clear discrimination in survival between male and female patients. Median survival time was 5 months in men and 10 months in women.

For validation of the nomogram, to guarantee that the model could be generally applied, and to avoid overfitting, it was necessary to evaluate the discrimination and calibration. We evaluated discrimination with the C-index, while calibration was typically surveyed by comparing the agreement between predicted and actual survival of patients as previously established.^[21]^ All of these comes about demonstrated that our nomogram had distant better separating and anticipating capacity than the conventional classification system. Beyond that, we also performed the decision curve analysis to study the clinical net benefit in Chinese SCLC patients’ prognosis of the nomogram^[22,23]^ and showed that this model improved the clinical net benefit across all threshold probabilities.

In addition, we constructed a system to group the patients to 2 levels of risk based on predicting total scores. In this consider, when the hazard stratification framework was connected in patients with the same T or N organization, it well discriminated the OS in each stage (Figs. 5A–I and 6A–I). For patients with T1 stage, although the significance was not reached (*P* = .073 and *P* = .055), we still saw a relatively clear trend in the Kaplan–Meier curve (Figs. 5B and 6B). A larger sample size is needed in further studies.

In summary, all of the results supported that this hazard stratification framework based on the nomogram was an accurate and reliable prognostic model. It seems to offer assistance to clinicians to distinguish the patients with tall hazards and to decide whether to perform the necessary adjuvant treatment on an individual level.

There were some certain limitations in our study. First and foremost, it was based on retrospective data, the limitation inherent to the SEER database. In spite of the fact that we performed a multivariate investigation to play down confounders related to the heterogeneities, the retrospective review nature of this ponder must be considered when translating the comes about. Moreover, there was possibly a variable selection bias when we designed the study. Third, the statistical analysis, as the data were partly derived from public data, did not give more detailed information on the specific drugs used for chemotherapy, the presence or absence of surgical treatment, and the presence or absence of comorbidities with other underlying diseases. Finally, the SEER database does not contain information about modern gene-array technology or a few vital atomic variables, such as PD-L1 expression,^[24]^ which have been demonstrated to be related to the in general survival of SCLC patients. Therefore, subsequent analysis of these aspects may improve the study design and the quality of the results within the near future.

## 5. Conclusion

We have developed and validated a novel nomogram for predicting survival in Chinese patients with initially metastatic SCLC. The required factors include demographic factors and clinicopathological characteristics, which are all easy to obtain, and the prognostic model is convenient to use. The nomogram provided clear prognostic superiority over the eighth edition of the AJCC-TNM staging system. It is the first model suitable for risk stratification in terms of long-term survival in Chinese SCLC patients. Thus, as an important supplement to the eighth edition of the AJCC-TNM staging system, combining the nomogram and risk stratification system could help clinicians to make individualized predictions of patients’ survival and to give necessary treatment recommendations. In addition, after surgical treatment of metastatic SCLC, chemotherapy in combination with other therapies is still the best current treatment option, especially after surgeons obtain tissues for pathologic analysis to arrive at tissue grading, which can further help surgeons to obtain more information to identify primary foci versus high-risk patients for the next treatment options.

## Acknowledgments

The authors recognize the endeavor of the Surveillance, Epidemiology, and End Results (SEER) Program tumor registries within the creation of the SEER database. The authors report no clashes of intrigued related to this work. Thanks to LetPub for its touch on the language and grammar of articles.

## Author contributions

**Data curation:** Rong Fu, Chuanqing Jing.

**Software:** Rong Fu.

**Conceptualization:** Chuanqing Jing.

**Writing – original draft:** Chuanqing Jing.

**Supervision:** Wei Zhang.

**Writing – review & editing:** Rong Fu, Wei Zhang.
